# Abstracts of the 2023 Pennsylvania College of Emergency Physicians Scientific Assembly

**DOI:** 10.1002/emp2.12946

**Published:** 2023-05-01

1

***2023 Pennsylvania College of Emergency Physicians Research Forum***

***May 5, 2023***

***Kalahari Resorts and Conventions ‐ Poconos***

***Pocono Manor, PA***

The 2023 Pennsylvania College of Emergency Physicians (PACEP) Research Forum is part of the annual Pennsylvania College of Emergency Physicians Scientific Assembly, May 4–6, 2023, at the Kalahari Resorts and Conventions in Pocono Manor, PA.

**2023 Pennsylvania College of Emergency Physicians (PACEP) Research Committee and Abstract Reviewers**

Eric Melnychuk, DO, FACEP, Research Committee Chair

Joseph Herres, DO, FACEP, Research Committee Co‐Chair

Alexandra Amaducci, DO

Bryan Kane, MD

Keith Willner, MD

Adam Sigal, MD, FACEP

Chadd Kraus, DO, DrPH, FACEP

Jan Reisinger, MBA, CAE

In accordance with the Accreditation Council for Continuing Medical Education (ACCME) Standards and the policy of the American College of Emergency Physicians, presenters must disclose the existence of significant financial interests in or relationships with manufacturers or commercial products that may have a direct interest in the subject matter of the presentation, and relationships with any commercial supporter of the Spivey Best of the Best plenary session, as the Pennsylvania College of Emergency Physicians Research Forum CME activity. These presenters do not consider that such relationships will influence their presentation.

No presenters had relevant disclosures to report. The authors also confirmed the abstracts submitted for publication have not been published, nor plan to be published elsewhere.

***William Spivey Research Abstract Sessions ‐ Friday May 5, 2023***

**Abstract Session #1**

**8:00am‐10:10am (Aloeswood Room, Kalahari Convention Center)**

1. The Impact of a Dedicated POCUS Program on FAST Compliance
*Heck M, Lehigh Valley Health Network*
2. Real Time Surveys Lead to Better Patient Satisfaction Scores
*Willner K, Geisinger Wyoming Valley Medical Center*
3. Compliance Analysis in VTE Prophylaxis: Harnessing Trauma Registry Data
*Mattiola R, Lehigh Valley Health Network*
4. Skin Glue: Does UV Light Cure it More Quickly?
*Nguyen M, St. Luke's University Health Network*
5. SBIRT: Brief Intervention Compliance and Validating Provider Screening
*Huang E, Lehigh Valley Health Network*
6. Development and Evaluation of a Novel Auricular Hematoma Task Trainer
*Levy C, Geisinger Medical Center*
7. How the COVID‐19 Pandemic Affected Providers of Different Job Titles Regarding Sleep Quality, Financial Situation, and Stress from Work
*Palladino A, St. Luke's University Health Network*
8. Reducing STEMI Door to Balloon (D2B) Time Using NEAR Criteria and Prehospital EKG Transmission
*Gopal J, Lehigh Valley Health Network*
9. Emergency Department Use and Uncompensated Care Following Medicaid Expansion in Pennsylvania
*Klaus A, Geisinger Commonwealth School of Medicine*
10. HER (History, ECG, Risk Factors) Scoring for Cardiac Risk Stratification in Patients <45 Years of Age Presenting with Chest Pain
*Legare C, St. Luke's University Health Network*
11. Sex Differences in Door to Balloon Time with Prehospital Activation: Is There a Difference?
*Patel A, Lehigh Valley Health Network*
12. Augmentation of Peripheral Venous Diameter for Ultrasound‐Guided Peripheral Intravenous Line Insertion
*O'Brien K, St. Luke's University Health Network*
13. Unilateral Versus Bilateral Traumatic Subarachnoid Hemorrhage and Patient Outcomes
*Rinaldi R, Geisinger Commonwealth School of Medicine*
14. Febrile Young Infants Age < = 60d with COVID‐19 Infection: Review of Clinical Presentation, Inflammatory Markers, Hospitalization Rate and Clinical Outcomes
*Yaeger S, Lehigh Valley Health Network*
15. Development and Evaluation of a Novel Peritonsillar Abscess Task Trainer
*Allen H, Geisinger Medical Center*
16. The Importance of Emphasizing Addiction Medicine During Medical Toxicology Fellowship Training: A Case Study of Tertiary Care Hospital System Toxicology Consultation Service
*Jones C, Lehigh Valley Health Network*
17. Pre‐hospital End Tidal Capnography as a Predictor for In‐hospital Resource Utilization and Mortality in Trauma Patients ‐ A Systematic Review
*Dew J, Guthrie Robert Packer*
18. PI Pathways for Monitoring Compliance in Trauma Resuscitation Management
*Huang E, Lehigh Valley Health Network*
19. System and Patient Characteristics Associated with Trauma Mortality in Pennsylvania
*Johnson C, Geisinger Commonwealth School of Medicine*
20. Performance Improvement and VNM Compliance: A Multidisciplinary Effort
*Mattiola R, Lehigh Valley Health Network*

**Abstract Session #2**

**10:30am‐11:30am (Aloeswood Room, Kalahari Convention Center)**

21. COVID‐19 Effects on Cardiac Arrest Care in Pennsylvania
*Reed‐Schrader E, Geisinger Wyoming Valley Medical Center*
22. Assessing Safety Outcomes of Patients Discharged from the ED after Receiving Phenobarbital for Alcohol Withdrawal
*Ebeling‐Koning E, Lehigh Valley Health Network*
23. How the COVID‐19 Pandemic Affected Different Specialties when it came to Sleep Quality
*Palladino A, St. Luke's University Health Network*
24. Intentional Self‐Harm Overdoses Before and During the COVID‐19 Pandemic in Adolescents Managed by a Medical Toxicology Consultation Service
*Gaetani S, Lehigh Valley Health Network*
25. Early Findings and Barriers from the Construction of a Patient‐Empowering Implementation Framework in the Emergency Department
*Vyas N, Geisinger Medical Center*
26. Intravenous Acetaminophen May Reduce Opioid Use for Pain in the Emergency Department
*DelBianco J, Lehigh Valley Health Network*
27. How the COVID‐19 Pandemic Affected Different Ages among Providers Regarding Perceived Stress level in the Workplace
*Palladino A, St. Luke's University Health Network*
28. Agents of Exposure among Pediatric Transgender Patients: An Analysis of the Toxicology Investigator's Consortium (ToxIC) Registry
*Ebeling‐Koning N, Lehigh Valley Health Network*
29. Evaluation of Emergency Department Patients with Isolated Traumatic Subarachnoid Hemorrhage
*Mileto A, Geisinger Commonwealth School of Medicine*
30. Expanded Use of Point of Care Ultrasound Curriculum for Attending Physicians
*Schultz K, Lehigh Valley Health Network*

**Plenary Session: William Spivey *Best of the Best* Presentations (Kalahari Ballroom, Kalahari Convention Center)**

**12:00pm‐1:00pm**

31. A Five‐Year Analysis of the Toxicology Investigators Consortium (ToxIC) Core Registry: Descriptive Differences among Patients who Identified as Transgender Compared to Cisgender, 2017–2021
*Beauchamp G, Lehigh Valley Health Network*
32. EMS Clinical Dashboard: Analyzing the Prehospital Care of Patients
*Huang E, Lehigh Valley Health Network*
33. Effect of Individualized Resident Throughput Metric Reports on Time to Disposition
*Golden B, Wellspan York Hospital*
34. Reduction in Computed Tomography Pulmonary Angiography (CTPA) Utilization in Pregnant Patients Suspected of Pulmonary Embolism via Pregnancy‐Adapted YEARS Criteria
*Krouse B, Geisinger Commonwealth School of Medicine*
35. First Attempt Success Between Anatomically and Physiologically Difficult Airways in the Emergency Department
*Herrera O, Allegheny Health Network*
36. Chest Pain in the Emergency Department: Does Race or Gender Influence whether a Patient gets a Cardiology Consult
*Greco D, St. Luke's University Hospital Network*

**Research Forum** 1 **Abstracts**

## 1 | The Impact of a Dedicated POCUS Program on FAST Compliance

**Heck M, Huff M, Harrison B, Bear E, Sharkazy J, Kane B, Wheel K, Stirparo**

**Lehigh Valley Health Network, Bethlehem, PA**

**Study Objectives**: Point‐of‐care ultrasound (POCUS) is a convenient imaging tool that has numerous applications for patient care. Our health system has 2 out of only 47 hospitals who are nationally accredited in Clinical Ultrasound by the American College of Emergency Physicians. In Pennsylvania, there are only 6 other hospitals that have been awarded this designation. We aimed to determine the effect of a dedicated POCUS ultrasound team during trauma evaluation and resuscitation at our Trauma 2 center.

**Methods**: We reviewed trauma alerts from January to December 2021 to determine the effect of our dedicated POCUS team during trauma resuscitation. Training is managed through emergency department (ED) bylaws and adheres strictly to the requirements for accreditation by the American College of Emergency Medicine's Clinical Ultrasound Board of Governors. Specific PI indicators included a Focused Assessment with Sonography for Trauma (FAST) examination not completed, image delays (computed tomography [CT] >30 min from arrival), chest x‐ray (CXR) not performed in the trauma bay, and the failure to complete a primary or secondary survey or untimely completion of a complete survey. FAST exam documentation was similarly reviewed for accuracy.

**Results**: From January 2021 to December 2021, there were 587 alerts, including 106 level 1, 468 level 2, and 13 pediatric trauma alerts trauma alerts. Our random sampling (goal 10 per month, see Table 1) found an overall FAST exam compliance of 99% with 98% accuracy. There was a single episode of documentation noncompliance in the first month of program implementation. Any inaccuracy in documentation was individually reviewed and re‐education was given as needed. Only one technological error occurred with a single incident of failure to upload FAST images. We continue to review FAST compliance on a quarterly basis with continuous review for any mortality. Additional interdisciplinary education is conducted during the Department of Emergency Medicine Grand Rounds.

**TABLE 1:** Random sample of monthly FAST documentation, compliance, and accuracy for year 2021.

Month	Number Reviewed	Documentation Completed	Compliance	Accuracy
**January**	10	9	90%	100%
**February**	10	10	100%	90%
**March**	10	10	100%	100%
**April**	10	10	100%	100%
**May**	2	2	100%	100%
**June**	9	9	100%	100%
**July**	10	10	100%	100%
**August**	10	10	100%	100%
**September**	10	10	100%	90%
**October**	1	1	100%	100%
**November**	15	15	100%	93%
**December**	1	1	100%	100%
**Total/Average**	**98**	**97**	**99%**	**98%**

Abbreviation: FAST, Focused Assessment with Sonography for Trauma.

**Conclusion**: From confirming a diagnosis, to facilitating safe and effective procedures through superior visualization of a patient's anatomy, ultrasound is a versatile tool that should be used in the care of trauma patents. While ultrasound is a mainstay in the trauma bay for FAST exams, we found that a dedicated POCUS team resulted in overall improvements in FAST exam speed, documentation, and protocol compliance. Future efforts will focus on expanding our current program to other hospitals in our network.

## 2 | Real Time Surveys Lead to Better Patient Satisfaction Scores

**Willner K**

**Geisinger Wyoming Valley Medical Center, Wilkes‐Barre, PA**

**Study Objectives**: Emergency physicians have a love/hate relationship with patient satisfaction surveys. Patients who are satisfied are more likely to adhere to the treatment plan and less likely to pursue legal action. However the current surveys are susceptible to recall and implicit bias. This study uses an analysis of data collected in a separate study to assess how key questions in the Doctors section of the Press Ganey (PG) survey compared to responses obtained by a trained volunteer in‐person.

**Methods**: This is an analysis of prospectively collected data obtained in a separate study evaluating how patients experience their emergency care when learners are present. Trained medical student volunteers administered the survey to a convenience sample of patients slated for discharge at a single community tertiary care hospital for a total of 12 weeks between June 2022 to October 2022. We compared this with the hospital's PG data for the questions on which the survey was based (Table 1).

**TABLE 1:** Responses to survey questions delivered in‐person versus by mail.

Question	In‐Person Survey Score (N)	Press Ganey Score (N)	P‐value
I felt informed about my treatment and diagnosis after my visit.	79.2 (262)	75.57 (265)	0.015
I felt like my provider(s) took time to listen to me during my visit.	84.98 (261)	79.6 (266)	0.047
How would you rate your satisfaction with your care team on this visit?	83.03 (263)	74.67 (265)	0.0013

**Results**: A total of 625 patients were approached over the study period with 313 agreeing to participate (response rate 50.1%). During the contemporaneous PG study quarter, the emergency department (ED) received 266 responses during the shifts for which the study enrolled patients, out of a total of 8460 discharged from the ED during those times (response rate 3.14%). All key questions favored the in‐person survey (N) vs. mailed PG (N): “I felt informed” score 79.2 (262) vs. 75.6 (265), p = 0.015; “I felt like my provider took time to listen” 85.0 (261) vs. 79.6 (266), p = 0.047; and “satisfaction with care team” 83.0 (263) vs. 74.7 (265), p = 0.0013.

**Conclusion**: This study shows a dramatically improved response rate to an in‐person survey compared with mail in PG forms, increasing its validity and making data less susceptible to recall bias. Results of the key questions compare favorably. A 5‐point difference in PG score could lead to a 30‐point change in percentile rank. This is a limited, single site study whose results are hypothesis‐generating but suggest a new pursuit for administrations seeking to truly understand their patients’ experience of their care.

## 3 | Compliance Analysis in VTE Prophylaxis: Harnessing Trauma Registry Data

**Mattiola R, Huff M, Harrison B, Bear E, Sharkazy J, Kane B, Wheel K, Stirparo**

**Lehigh Valley Health Network, Bethlehem, PA**

**Study Objectives**: The prevention of thromboembolic events with appropriate venous thromboembolism (VTE) prophylaxis is paramount in the care of trauma patients, as all trauma patients are at an elevated risk for VTE. Given the absolute necessity of VTE prophylaxis in the setting of trauma, deviations from 100% compliance place patients at an unacceptably high risk for life‐threatening VTE such as pulmonary embolism. We aimed to determine the overall rate of appropriate VTE prophylaxis for trauma patients at a Level 2 Adult Trauma Center by leveraging existing trauma registry data.

**Methods**: We reviewed all encounters through the retrospective review of trauma registry data from January 2021 to December 2021 for the appropriate administration of VTE prophylaxis (Figure 1). Our primary chemoprophylactic agent was enoxaparin with dosing adjustments as needed for weight. We introduced a forced function into patient documentation, as holding VTE prophylaxis required physician‐to‐physician discussion and a specific order placed into the electronic medical record (EMR). Fallouts were individually recorded and reviewed by our dedicated trauma performance improvement team. A documentation adjunct was created for the electronic medical record (EPIC) with a specific EHMR dot phrase which was integrated into our standardized Trauma H&P template. Weekly compliance was reviewed with group education given to all trauma clinicians. Additional education was given on an as‐needed individual basis.

**FIGURE 1:**
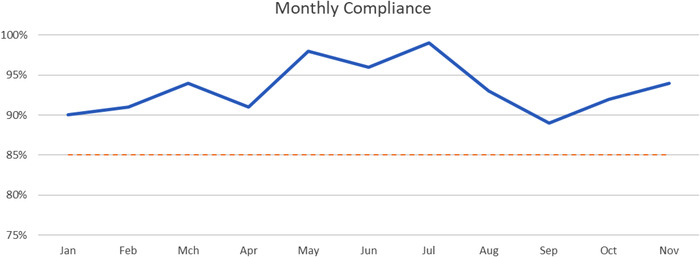
Overall monthly compliance with VTE prophylaxis for year 2021. Abbreviation: VTE, venous thromboembolism.

**Results**: In total, protocol adherence for VTE prophylaxis was reviewed for 969 patients with 63 cases of noncompliance, resulting in an overall compliance rate of 93.5%. The distribution of VTE chemoprophylaxis type was 70.1% low molecular weight heparin (LMWH), 9.3% subcutaneous heparin, 8.4% factor Xa inhibitors, and 12.2% other agents. The average time to initiation of chemoprophylaxis was 16.61 hours with 44.6% occurring within 12 hours and 73.5% of total encounters beginning in the first 24 hours. Only three total adverse events occurred over the 12‐month study period, with 2 cases of deep‐vein thrombosis and 1 case of pulmonary embolism.

**Conclusion**: The application of trauma registry data by individual institutions is a powerful tool for maximizing program improvement and evaluating granular and aggregate protocol adherence. Our utilization of trauma registry data for the evaluation of VTE prophylaxis initiation led to significant improvements in our rates of compliance and minimal of adverse events.

## 4 | Skin Glue: Does Ultraviolet Light Cure it More Quickly?

**Nguyen M, Sterner S, Stoltzfus J, Balakrishnan V, Stankewicz H**

**St. Luke's University Hospital Network, Easton, PA**

**Study Objectives**: Topical skin glue is used frequently in the emergency department to repair lacerations and to close incisions used for paracentesis or thoracentesis. It takes time for the skin glue to cure. If an intervention such as ultraviolet (UV) light could decrease the time it takes to cure the skin glue, it would be beneficial. Situations with pediatric patients would benefit as they would need to sit still or be restrained for less time and would be able to touch the skin glue quicker. For critically ill patients, it would help to decrease unnecessary time waiting for glue to cure after procedures. Additionally, it could help decrease length of stay times for patients in the emergency department.

**Methods**: This will be a prospective study using healthy volunteers who will have two 4 cm linear applications of skin glue placed on their forearm. One will have UV light applied to it (at 3–5 cm from skin) for 5 second intervals and after each interval the subject and 1 blinded investigator will mark on a visual analogue scale the “stickiness” of the glue. This will continue every 5 seconds until the glue is cured. The other line will have no intervention and at 5 second intervals the subject and a blinded investigator will mark on a visual analogue scale the “stickiness” of the glue. This will continue every 5 seconds until the glue is cured. The interventions will be randomly assigned and only the investigator administering the interventions will know which is being used. A barrier will be used to blind the other investigator and subject and prevent them from seeing which intervention is being used. Lastly, the subject will be asked which intervention they were most satisfied with and if there were any complaints or issues with the interventions.

**Results**: The average curing time for the control skin glue group was 78.1 seconds, while the average curing time for the UV light group was 75.4 seconds when judged by the subject. The average curing time for the control skin glue group was 76.1 seconds, while the average curing time for the UV light group was 73.8 seconds when judged by the subject. There was no difference in patient satisfaction.

**Conclusion**: There is no statistical significance between the UV light group and the control group in average time for skin glue curing. Despite some ED physicians believing UV light decreases time to curing, this was not found to be true in this study. Based on these findings, there is no benefit to using UV light when using skin glue.

## 5 | Screening, Brief Intervention, and Referral to Treatment (SBIRT): Brief Intervention Compliance and Validating Provider Screening

**Huang E, Huff M, Harrison B, Bear E, Sharkazy J, Kane B, Wheel K, Stirparo**

**Lehigh Valley Health Network, Bethlehem, PA**

**Study Objectives**: All trauma patients should be screened for substance use disorder and a high index of suspicion should be maintained for all types of substance use disorders (SUD). Screening, Brief Intervention, and Referral to Treatment (SBIRT) is an effective protocol for the early intervention and treatment of individuals with substance use disorders or those at risk of developing SUDs. While we were meeting the Pennsylvania Trauma Systems Foundation (PSTF) 80% requirement for substance abuse screening, we identified that substance abuse counseling compliance was not meeting our internally set expectation of 90%.

**Methods**: We created a protocol for evaluating and screening patients for SUD. The protocol was integrated into our electronic health record (EHR) (EPIC) with standardized dot‐phrases, which were introduced with alterations to the departmental progress note. In addition to a positive provider screen, indications for urine drug screen or ETOH included altered mental status, unconsciousness, conscious with odor of alcohol, history of substance abuse, presence of stigmata of drug abuse, dilated or constricted pupils, nystagmus, and provider discretion. Our interventions included Clinical Institute Withdrawal Assessment (CIWA) or Clinical Opiate Withdrawal Scale (COWS) as appropriate, substance abuse counseling (Psychiatry), consult to toxicology, and social work.

**Results**: From July 2020 to December 2020, there were 390 trauma patients evaluated. Substance abuse screening was performed in 95.90% of patients (Figure 1). Of those with a positive screen, substance abuse counseling was completed in 78.05% of patients. Following our intervention, from January 2021 to December 2021, there were 893 trauma patients seen. Substance abuse screening was completed in 887 and not completed for 6, for an overall compliance of 99.33% (Figure 2). Substance abuse counseling was positive in 158 and substance abuse counseling (SAC) was completed in 152 for an overall compliance of 96.20%. Overall, we saw increases in both the rates of substance abuse screening and counseling after our intervention.

**FIGURE 1:**
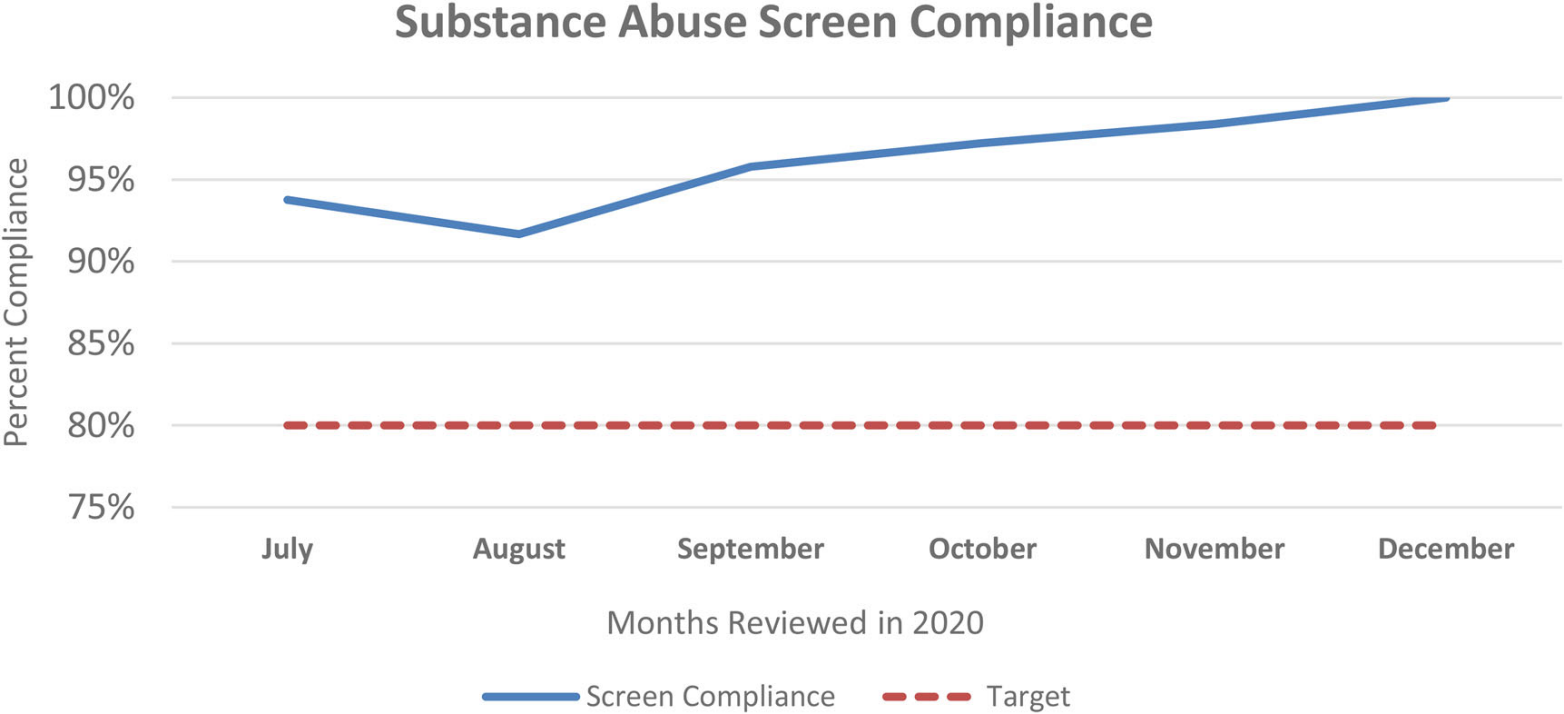
Performance on substance abuse screening from July to December 2020 compared to the recommended target of 80%.

**FIGURE 2:**
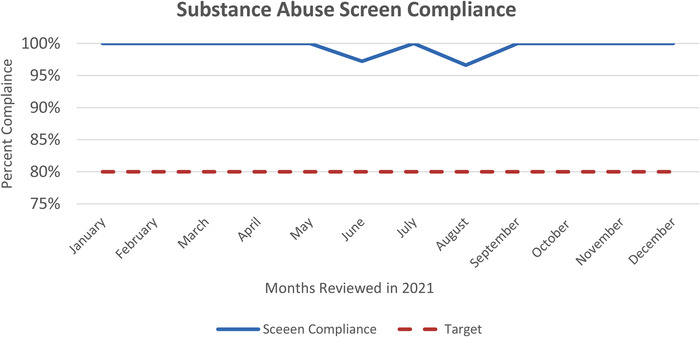
Performance on substance abuse screening from January to December 2021 compared to the recommended target of 80%.

**Conclusion**: Continuous re‐evaluation of program success indicated that sustained reinforcement of the need for screening and substance abuse counseling was necessary for continued benefit. We provided individual feedback to individual providers when needed as well as general group education sessions regarding substance abuse screening and counseling. Data was analyzed monthly with weekly noncompliance reviewed at our weekly performance improvement meetings.

## 6 | Development and Evaluation of a Novel Auricular Hematoma Task Trainer

**Levy C, Senter J, Senter N, Melnychuk E**

**Geisinger Medical Center, Danville, PA**

**Study Objectives**: Head and neck trauma are common complaints evaluated in the emergency department. Auricular hematomas are seen and managed in the emergency department (ED). However, there are no standard task trainers for management of an auricular hematoma. A task trainer for the management of auricular hematomas was created and subsequently evaluated by emergency medicine trainees, to augment learning related to the management of auricular hematomas.

**Methods**: Surgical skin pads from procedural simulation mannequins were traced with a white charcoal pencil using an ear stencil, and then cut out from the pad. A knife blade was then used to make a perpendicular incision on the edge of the material and the space was widened between the two layers of the separated material. A piece of 2×2 cotton gauze was cut in half and placed into the pre‐made incision to simulate a hematoma. The simulated ear was then tacked to a Styrofoam head with pushpins for use. A lecture on management of auricular hematomas was given prior to trainees simulating the procedure of incision and drainage of an auricular hematoma. The subcutaneous surgical skin pads (which created 10 reusable ears) and polystyrene foam head had an estimated cost of less than $100 if purchased new. A pre‐ and post‐procedural simulation survey was created to identify current level of confidence with management of auricular hematomas and utility of the task trainer. Review by the institutional institutional review board (IRB) determined this project to be exempt.

**Results**: Pre‐simulation survey using a 5‐point Likert scale somewhat average lower confidence levels in managing an auricular hematoma prior to the simulation (2.1/5), which improved after the lecture and drainage simulation (4.23/5). Trainees thought a task trainer would teach the necessary procedural skills (4.75/5) and after using the task trainer, found the task trainer to be adequate (4.69/5). Qualitative evaluation of comments written on the post‐survey found most all comments to be positive with mention of it being helpful. One comment suggested using more realistic ears.

**Conclusion**: Development of an auricular hematoma task trainer with available equipment was feasible. Based on structured feedback, using an auricular hematoma task trainer is a suitable strategy to augment learning and skill development for management of auricular hematomas in emergency medicine trainees.

## 7 | How the COVID‐19 Pandemic Affected Providers of Different Job Titles Regarding Sleep Quality, Financial Situation, and Stress from Work

**Palladino A, Deeb M, Stoltzfus J, Morley K, Patterson R, Stankewicz H**

**St. Luke's University Health Network, Easton, PA**

**Study Objectives**: The COVID‐19 pandemic affected the overall wellness of health care professionals worldwide. This study provides insight into how health care professionals’ wellness was impacted throughout the course of the pandemic, whether positive or negative. The focus of this study relies on comparing the pandemic's impact on wellness of various job titles. Since the wellness of the healthcare team is important for the holistic care of the patient and their loved ones, it is important to assess the effects of the pandemic on their wellness.

**Methods**: The study was conducted through a survey via survey monkey to health care professionals of a healthcare network in eastern Pennsylvania. The study was sent to residents in training, fellows, nurses, attending physicians, as well as advanced practitioners in specialties including Emergency Medicine, Family Medicine, Internal Medicine, and Critical Care. The survey was sent on January 11th, 2022 and remained open over a 2‐week period. All data collected in this study was anonymous. The responses to the survey questions about wellness were measured as 1 being not at all true to 5 being completely true. Several categories were compared to distinguish the providers from one another in addition to specialty, including age, with whom the provider lives, and the average number of clinical hours worked a month. Collectively, there were 267 surveys received of which 125 were male, 141 were female, and 1 prefered not to identify. 34 were Critical care, 147 were Emergency Medicine, 28 were Family Medicine, and 48 were Internal Medicine. 63 were Residents, 3 were Fellows, 34 were Nurses, 101 were Attending Physicians and 59 were NP/PAs.

**Results**: Results showed a difference in responses to four of the questions on the survey including “I have trouble falling asleep,” “I have trouble staying asleep,” “I am concerned about my financial situation,” and “I am stressed at work.” We observed a significant difference to each of these questions (See Figure 1). Regarding trouble falling asleep, residents in training versus NPs/PAs showed a p‐value of .002 while attending physicians versus NPs/PAs showed a p‐value of .004. For professionals having trouble staying asleep, there was a significant difference among residents in training versus NPs/PAs with a p‐value of .002. There was also a significant difference in the question pertaining to concern about one's financial situation where residents in training versus attending physicians show a p‐value <.001, residents in training versus NPs/PAs show a p‐value <.001, nurses versus attending physicians show a p‐value <.001, and nurses versus NPs/PAs show a p‐value of .001.

**FIGURE 1:**
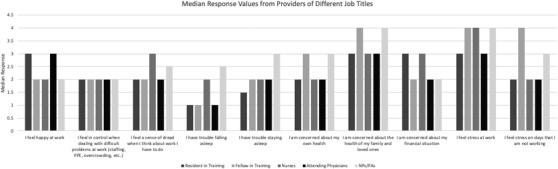
Median response values from healthcare professionals of different job titles.

**Conclusion**: Sleep, financial concern, and stress from work can all contribute to a provider's care in the workplace. NPs/PAs suffer significantly greater than residents in training and attending physicians regarding having trouble falling asleep over the course of the COVID‐19 pandemic. NPs/PAs also have a harder time staying asleep when compared to residents in training. When assessing a provider's financial concern, residents in training are more concerned than attending physicians and NPs/PAs, and nurses are also more concerned when compared to attending physicians and NPs/PAs. Finally, residents in training feel less stress at work than NPs/PAs. The wellness questionnaires allow us to assess the impact of the pandemic on providers across several job titles; our results contain a wide range of areas marking concern for wellness of providers, and this warrants further research into why or how these differences arise.

## 10 | HER (History, ECG, Risk Factors) Scoring for Cardiac Risk Stratification in Patients <45 Years of Age Presenting with Chest Pain

**Legare C, Arner K, Diaz T, Ridley K, Stankewicz H, Jeanmonod R**

**St. Luke's University Hospital Network, Easton, PA**

**Study Objectives**: Ischemic heart disease is a leading cause of death among adults in the United States. Approximately 90% of young people diagnosed with acute myocardial ischemia (AMI) present with chest pain. Ultimately, only 13% of patients presenting with chest pain have acute coronary syndrome (ACS). The HEART (History, ECG, Age, Risk Factors, Troponin) score is a validated risk stratification tool for this patient population; however, bloodwork for troponin testing is required and a delta is commonly necessary. The purpose of this study was to determine the utility of using the (History, ECG, Risk Factors) HER components of the HEART score for the rule‐out of 30‐day Major Adverse Cardiac Event (MACE), ACS, ventricular tachycardia, and ventricular fibrillation in patients aged less than 45 to promote improved resource allocation, ED crowding, and patient satisfaction. Additionally, given the vulnerability of MACE to workup bias and how troponin testing is a commonly used endpoint for disposition planning in the emergency department, we investigated the predictive potential of HER scoring for troponin positivity.

**Methods**: This is a retrospective cohort study that examined a consecutive cohort of patients less than 45 years of age presenting to a single health network with a chief complaint of chest pain and documented HEART score between 1/2019 and 12/2020. Ultimately, between automatically pulled and manually reviewed data, information regarding patient HEART score, HER score, HER components, demographics, emergency department disposition, 30‐day MACE, 30‐day mortality, troponin positivity, catheterization results, and encounter final diagnosis were analyzed. HER scores ≤1 were categorized as negative and scores of ≥2 were categorized as positive. Sensitivity, specificity, and predictive values were calculated for the relationship between HER score positivity and troponin results.

**Results**: 7935 patients, under the age of 45 years, were enrolled. 107 were excluded as they were <18 years old and 104 were not included because a troponin was not checked. The population was 47.4% male and 52.6% female. Age distribution, between the sexes, matched: median 35 (IQR 29–40). Men were found to have significantly higher HEART scores (p<0.0001). 87.2% of patients were discharged and 9.9% were admitted. None of the patients who were discharged, transferred to other services, or left against medical advice were found to have mortality or MACE at 30 days. 87 of the admitted patients underwent catheterization: five were found to have coronary artery disease. In this group, all had HER scores >1. Overall, 1.1% of patients were diagnosed with ACS, Vtach, Vfib, cardiac arrest. More patients with positive troponin testing had non‐ACS related disease. The prevalence of troponin positivity was 1.7%. The test characteristics of HER scoring for troponin positivity were: sensitivity 80 (71.9‐86); specificity 71.3 (70.3‐72.3); PPV 4.7; NPV 99.5.

**Conclusion**: These data suggest that troponin testing is low‐yield in the risk stratification of patients under the age of 45 years for acute coronary syndrome and 30‐day MACE. However, this test likely has a role in the diagnosis and risk stratification of other disease processes.

## 12 | Augmentation of Peripheral Venous Diameter for Ultrasound‐Guided Peripheral Intravenous Line Insertion

**O'Brien K, Ciccarelli B, Reiss A, Balakrishnan V, Stankewicz H**

**St. Luke's University Hospital Network, Easton, PA**

**Study Objectives**: Ultrasound‐guided intravenous line placement is utilized often in the emergency department for venous access in patients whose veins are difficult to cannulate by traditional methods. This study aims to identify specific interventions, using readily available devices found in most emergency departments, that may augment venous cross‐sectional area.

**Methods**: 41 resident and medical student volunteers had their basilic vein identified on ultrasound, specifically 5 centimeters proximal from the antecubital fossa. At this point, the diameter (vertical and horizontal) were measured with no intervention (ie, no tourniquet) with the arm positioned parallel to the floor as well as approximately 45 degrees below the level of the bed. These two positions were repeated with the following interventions: one rubber tourniquet applied approximately 5 cm proximal to where the vein measurement was done, an additional rubber tourniquet applied 5 cm proximal to first tourniquet, blood pressure cuff inflated to between 160 to 200 mmHg, CAT battle tourniquet application, and soaked warm towel applied to brachium for up to one minute. The primary outcome of this study was to evaluate the increase in venous cross‐sectional area from baseline measurement.

**Results**: All interventions were statistically significant in increasing venous cross‐sectional area as compared to no intervention, with the most significant being CAT battle tourniquet (p < 0.001, mean change +7.32 mm^2^, 95% confidence interval (CI) +5.73 to + 8.91 mm^2^). The change in position of the arm being measured was not statistically significant for any intervention except for CAT tourniquet (p = 0.0056, mean change ‐1.74 mm^2^, 95% CI –0.54 to –2.93 mm^2^). Notably, there was no significant difference between two tourniquets and blood pressure cuff (p = 0.496, mean change +0.58 mm^2^, 95% CI –1.13 to +2.29 mm^2^), but there was a significant increase in cross‐sectional area with CAT tourniquet use compared to blood pressure cuff (p = 0.018, mean change +1.62 mm^2^, 95% CI +0.29 to +2.95 mm^2^). Lastly, two tourniquets increased cross‐sectional area compared to one tourniquet (p < 0.001, mean change +2.20 mm^2^, 95% CI +1.14 to +3.26 mm^2^).

**Conclusion**: This study identified several potential interventions for maximizing venous cross‐sectional area on ultrasound which we believe would likely translate to an increased success rate with USG peripheral IV insertion. All the tested interventions resulted in statistically significant increases in cross‐sectional area. Arm positioning (neutral or below the level of the bed) did not show significant changes in most interventions, with the exception of the CAT tourniquet. The devices used in this study are readily accessible in most emergency departments, allowing the results to be applicable to most practicing emergency physicians. Further study should be performed on how these maneuvers affect success in ultrasound‐guided intravenous line placement.

## 14 | Febrile Young Infants Age ≤60 days with COVID‐19 infection: Review of Clinical Presentation, Inflammatory Markers, Hospitalization Rate and Clinical Outcomes

**Mori M, Villalobos T, Yaeger S, Busse C**

**Lehigh Valley Health Network, Bethlehem, PA**

**Study Objectives**: A risk‐stratified approach to the evaluation and management of febrile young infants < 60 days old based on inflammatory markers (IM) has been promoted by several recent publications including the 2021 national American Academy of Pediatrics guideline. Such risk‐stratified protocols decrease need for invasive work‐up, hospitalization, and empiric antibiotic use. Procalcitonin (PCT) has emerged as the premier IM to identify infants at high risk for invasive bacterial infection. During the COVID‐19 pandemic, however, the predictive value of PCT has been called into question by studies on adult patients with elevated PCT during COVID‐19 infection. We aimed to describe retrospectively the clinical characteristics and PCT as an inflammatory marker in febrile young infants with COVID‐19.

**Methods**: Our regional health network serves approximately 40,000 pediatric emergency visits per year in five general and one pediatric emergency rooms. From April 2020 to September 2022, 166 infants age ≤ 60 days presented with a chief complaint of fever. Previous work had established a clinical pathway with PCT‐based risk stratification to guide invasive work‐up and hospitalization. Charts without documented PCT value were excluded from this retrospective analysis.

**Results**: There were 47/166 (28%) febrile infants that had a positive COVID‐19 test. Of those, 36/47 (77%) had a documented PCT value (see Table 1). Of the reviewed cases, 22/36 (61%) presented with upper respiratory symptoms. The most common symptom was gastrointestinal, 10/36 (28%) followed by 6/36 (17%) with lower respiratory symptoms, and 7/36 (19%) had a combination of respiratory and gastrointestinal symptoms. None of the COVID+ infants were found to have an invasive bacterial infection (IBI). Six positive blood cultures were deemed contaminants. The inflammatory markers PCT and white blood count (WBC) were consistently found within normal limits. The absolute neutrophil count showed large variability. Only 7/36 (19%) patients underwent lumbar puncture. The majority 25/36 (69%) of patients were hospitalized with an average length of stay < 2 days. Three patients returned to emergency department for evaluation of persistent fever.

**TABLE 1:** COVID‐19+ febrile infants aged 0–60 days, April 2020 thru September 2022.

**Variable (N = 36)**	**Results**	**Comments**
Demographics Female/Male Age White/Caucasian Hispanic/Latino African American/Black Multiracial Unknown	22/14 (61%/39%) 8–60 days (average 29 days) 12 (33%) 9 (25%) 5 (14%) 6 (17%) 3 (8%)	
Max temperature documented	100‐102°F (average 100.7°F)	
Invasive work‐up (Lumbar Puncture)	7/36 (19%)	
Procalcitonin (ng/dl)	0.05‐0.72 ng/dl	
White blood cell count	2.3‐15.6 K/cmm	
Absolute neutrophil count	396‐6700/cmm	
Invasive bacterial infection	0/36 (0%)	6 contaminant blood cultures *S. salivarius, S. epidermidis, S. hominis, S. mitis*
Hospitalized	25/36 (69%)	
Length of stay	11‐91 hours (average 29 hours)	
Return ED visit or readmission	3 (8%)	Recurrence of fever

Abbreviation: ED, emergency department.

**Conclusion**: This series of 36 COVID‐19 positive infants with documented IMs validates a previously established PCT‐based risk‐stratified approach to the evaluation and management of febrile young infants. During the pandemic, a substantial proportion of febrile infant were found to have COVID‐19. Our results suggest that PCT is not elevated in young infants with COVID‐19. The predominance of mild upper respiratory symptoms, low hospitalization rate and short LOS indicate low illness severity in infants. There were no cases of concomitant IBI. The main limitation of this descriptive study is the small sample size. Our review intentionally selected COVID‐19 patients to investigate early patterns of PCT in relation to invasive bacterial infection. Further investigation of this relationship is warranted with a larger sample size to characterize inflammatory markers in COVID‐19 vs other viral infections and IBIs.

## 15 | Development and Evaluation of a Novel Peritonsillar Abscess Task Trainer

**Allen H, Pichardo P, Wilson C, Melnychuk E**

**Geisinger Medical Center, Danville, PA**

**Study Objectives**: Peritonsillar abscesses are the most common deep infection of the head and neck and often prompts emergency department evaluation. Drainage of this type of abscess in the emergency department is a skill needed by emergency medicine physicians. There are few published peritonsillar abscess task trainers and no known commercial task trainers known to the authors. With readily available materials, the authors developed and evaluated a task trainer to augment learning related to peritonsillar abscess management.

**Methods**: The head and neck of a legacy simulation mannequin was secured to a base. Arterial tubing filled with water dyed with yellow food coloring was coiled and placed behind the posterior pharynx to simulate a fluid collection. Cotton gauze was then placed behind the tubing to augment swelling in the posterior pharynx. A lecture on management of peritonsillar abscess was given prior to trainees simulating the procedure of drainage of a peritonsillar abscess. A pre‐ and post‐procedural simulation survey was created to identify current level of confidence with management of peritonsillar abscesses and utility of the task trainer. Review by the institutional IRB determined this project to be exempt.

**Results**: Pre‐simulation survey using a 5‐point Likert scale somewhat average lower confidence levels in managing a peritonsillar abscess prior to the simulation (2/5), which improved after the lecture and drainage simulation (4.18/5). Trainees thought a task trainer would teach the necessary procedural skills (4.86/5) and after using the task trainer, found it to be adequate (4.56/5). Qualitative evaluation of comments written on the post‐survey found all comments to be positive with mention of improving confidence.

**Conclusion**: Development of a peritonsillar abscess task trainer with available legacy equipment was feasible. Based on structured feedback, using a peritonsillar abscess task trainer is a suitable strategy to augment learning and skill development for management of peritonsillar abscesses in emergency medicine trainees.

## 18 | PI Pathways for Monitoring Compliance in Trauma Resuscitation Management

**Huang E, Huff M, Harrison B, Bear E, Sharkazy J, Kane B, Wheel K, Stirparo**

**Lehigh Valley Health Network, Bethlehem, PA**

**Study Objectives**: The acute evaluation and resuscitation of trauma patients should strictly adhere to Advanced Trauma Life Support (ATLS) principles. Streamlined institutional protocols and continuous training are essential to the continued delivery of excellent patient care. We aimed to review our trauma resuscitation management performance at our Trauma 2 center to ensure appropriate protocol adherence.

**Methods**: We reviewed all trauma alerts from January 2021 to December 2022 to assess overall compliance in our trauma resuscitation protocols. Our protocol included uninterrupted emergency medical services (EMS) time for handoff, strict adherence to ATLS principles, and a rapid response time by the on‐site trauma surgeon. Key performance indicators included airway evaluation documentation, airway management protocol adherence, trauma surgeon arrival time (<15min), triage, priority labs, ATLS deviation, and trauma surgeon backup utilization and timeliness. Interdisciplinary collaboration between Trauma and Emergency Medicine was facilitated during committee meetings and quarterly emergency department (ED) Grand Rounds presentations.

**Results**: Overall, there 587 alerts, including 106 level 1, 468 level 2, and 13 pediatric trauma alerts (Table 1). Airway evaluation documentation had an average compliance of 99% with only 2 episodes of noncompliance by two providers during the 12‐month period (Table 2). Our intubation protocol requires the use of a glideslope for endotracheal tube placement, the utilization of appropriate rapid sequence intubation (RSI) medications, involvement of an anesthesia physician after a second failed intubation attempt, and accurate documentation of all events. Intubation protocol compliance was 93%. Trauma surgeon response time (<15 min) compliance was 100% across all 587 alerts, greatly surpassing the required 80% threshold for Level 1 TTA. The compliance for prioritized laboratory results was 86%, requiring additional education to laboratory staff to accommodate acute trauma laboratory orders.

**TABLE 1:** Overview of trauma alert distribution from January to December 2021.

Month	Level 1 Alert	Level 2 Alert	Pediatric Alert
**January**	9	32	2
**February**	9	37	1
**March**	10	44	1
**April**	7	40	2
**May**	10	38	0
**June**	10	32	0
**July**	10	42	2
**August**	7	37	3
**September**	10	45	0
**October**	7	37	0
**November**	7	35	2
**December**	10	49	0
**Total**	**106**	**468**	**13**

**TABLE 2:** Trauma bay intubation protocol compliance from January to December 2021.

Month	Number of Intubations	Non‐Compliant	Compliance
**January**	1	0	100%
**February**	2	0	100%
**March**	1	0	100%
**April**	0	0	NA
**May**	1	0	100%
**June**	1	0	100%
**July**	0	0	NA
**August**	3	1	67%
**September**	0	0	NA
**October**	3	0	100%
**November**	2	0	100%
**December**	1	0	100%
**Total/Average**	**15**	**1**	**93%**

**Conclusion**: Prioritizing adherence to ATLS standards of care is essential in the safe and effective care of trauma patients in the acute setting. Collaboration between Trauma Surgery and Emergency Medicine teams should be prioritized in the development of clinical protocols.

## 19 | System and Patient Characteristics Associated with Trauma Mortality in Pennsylvania

**Johnson C, Shahan J, Kraus C, Kupas D, Lopez R**

**Geisinger Health System, Danville, PA**

**Study Objectives**: Pennsylvania (PA) has one of the oldest and most highly developed trauma systems in the United States (US), including a growing number of Level 3 and 4 trauma centers (TCs). The study examines system and patient characteristics associated with trauma mortality in PA.

**Methods**: Institutional review board (IRB)‐approved, retrospective study of mortality among adult (age ≥ 15 years) patients in the PA Trauma Systems Foundation Trauma Registry Data Set (PTOS) between 2016–2020. Primary outcome measure was death prior to hospital discharge. Mortality rates were analyzed by system and patient factors including: proximity to TC, mechanism of injury, age, and intensive care unit (ICU) length of stay.

**Results**: 222,247 records were analyzed. Among 156,813 patients injured in counties with a TC, mean age was 59.9 years (standard deviation [SD] 23.1), blunt injuries were most common (89%), mean total ICU days were 1.3 (SD 4.35), and 95.5% were alive at hospital discharge. In this group, for every 1‐year increase in age, odds of death increased 1.1% (95% confidence interval [CI] 1.010, 1.012). Patients with penetrating injuries had 6.5 times higher odds (95% CI 6.1, 6.9) of death versus those with blunt injuries. Every ICU day increased odds of death by 4.2% (95% CI 1.039, 1.046). Among 65,434 patients injured in counties without a TC, mean age was 56.2 years (SD 22.5), blunt injuries were most common (90.3%), mean total ICU days were 1.3 (SD 4.11), and 96.6% were alive at hospital discharge. In this group, for every 1‐year increase in age, the odds of death increased 3.8% (95% CI 1.035, 1.040). Patients with penetrating injuries had 3.1 times higher odds (95% CI 2.6, 3.7) of death versus those with blunt injuries, and every ICU day increased odds of death by 6% (95% CI 1.054, 1.067). 7,037 patients were initially treated at level 3 or 4 TC before transfer to level 1 or 2 TC. Mean age was 59.9 years (SD 22.4), blunt injuries were most common (94%), mean total ICU days were 1.2 (SD 3.5), and 97.6% were alive at hospital discharge. In this group, for every 1‐year increase in age, the odds of death increased by 3.2% (95% CI 1.023, 1.041), and every ICU day increased odds of death by 10.2% (95% CI 1.080, 1.124).

**Conclusion**: These results help inform efforts to optimize existing and future regionalized systems of trauma care. Further analysis will focus on additional characteristics to guide placement of TCs.

## 20 | Performance Improvement and Vitals Neuro‐Motor Compliance: A Multidisciplinary Effort

**Mattiola R, Huff M, Harrison B, Bear E, Sharkazy J, Kane B, Wheel K, Stirparo**

**Lehigh Valley Health Network, Bethlehem, PA**

**Study Objectives**: The Pennsylvania Trauma Systems Foundation (PTSF) issues trauma level accreditation for hospitals in the state of Pennsylvania. Nursing documentation of trauma patient vital signs taken within 10 minutes of arrival, recording of hourly trauma patient vital signs, and hourly neurological status documentation for trauma patients with suspected neck or head injury are some of the requirements for accreditation by PTSF. For these components, the goal is 80% compliance. We identified that our overall compliance was significantly under target and in need of corrective action.

**Methods**: Weekly retrospective chart reviews were performed from September 2021 through April 2022. Our inclusion criteria were all emergency department trauma patients >12 years old assessed via Level 1 or 2 trauma activation or via consult to the Trauma service. Percent compliance for vitals taken within 10 minutes of patient arrival (V10), neurological including documentation Glasgow Coma Scale (GCS) and motor documentation (NM) of all patients suspected of having head or neck injury, and hourly vitals (HV) was recorded. Collectively these are referred to as vitals neuro‐motor (VNM). Subset analysis of Level 1 and 2 trauma activations versus consult percent compliance was assessed.

**Results**: Several actions were subsequently initiated to improve compliance of nursing documentation in these areas. Our multifaceted plan included interdepartmental collaboration with the emergency department with real‐time quality improvement reviews, biweekly data review, feedback to noncompliant nurses, reward programs, daily sign‐out, review and education, online education, and dedicated core trauma nursing. Compliance for trauma alerts was consistently above benchmark (Table 1). We identified marked improvements in average overall compliance from September 2021 (V10 = 63%, NM = 64%, HV = 59%) versus April 2022 (V10 = 84%, N = 96%, HV = 81%).

**TABLE 1:** Trauma alert overall compliance percentages for vitals neuro‐motor (VNM) monitoring.

Week	Vital Signs Documented Within 10 Minutes of Arrival	Hourly Neuro‐Motor Documentation	Hourly Vital Sign Documentation
Sept 1‐Sept 5	100%	100%	100%
Sept 6‐Sept 12	80%	N/A	80%
Sept 13‐ Sept 19	83%	100%	100%
Sept 20‐ Sept 26	100%	100%	100%
**Monthly Compliance**	91%	100%	95%
Sept 27‐ Oct 3	100%	100%	100%
Oct 4‐ Oct 10	100%	100%	67%
Oct 11 ‐ Oct 17	100%	100%	100%
Oct 18 ‐ Oct 24	100%	50%	83%
Oct 25 ‐ Oct 31	75%	100%	100%
**Monthly Compliance**	95%	90%	90%
Nov 1 ‐ Nov 7	86%	0%	71%
Nov 8 ‐ Nov 14	100%	100%	100%
Nov 15 ‐ Nov 21	60%	100%	80%
Nov 22 ‐ Nov 28	100%	100%	75%
**Monthly Compliance**	87%	75%	82%
Nov 29 ‐ Dec 5	80%	100%	80%
Dec 6 ‐ Dec 12	100%	100%	100%
Dec 13 ‐ Dec 19	50%	100%	100%
Dec 20‐ Dec 26	100%	100%	100%
Dec 27‐ Dec 31	100%	N/A	100%
**Monthly Compliance**	86%	100%	96%
**Total Average**	**90%**	**91%**	**91%**

**Conclusion**: This multidisciplinary effort for improvement in patient care has resulted in a culture change highlighting the importance of ongoing documentation of vitals within 10 minutes, neuro, and hourly vitals. Ongoing weekly retrospective reviews and incorporation into dedicated registry components have aided in statistical analysis. We continue to support “Trauma Thursday's” which emphasize trauma education and VNM. Further, we are integrating additional multidisciplinary support with expanded roles for high‐performing Core Trauma RNs.

## 21 | COVID‐19 Effects on Cardiac Arrest Care in Pennsylvania

**Reed‐Schrader E, Mohney S, Willner K,**

**Geisinger Wyoming Valley Medical Center, Wilkes‐Barre, PA**

**Study Objectives**: COVID‐19 disrupted the healthcare system in unanticipated ways. Up to 40% of adults in the US avoided seeking care (1). International data reflects increased incidence of out of hospital cardiac arrest (OHCA), and lower rates of bystander interventions (1, 2). We sought to determine if the incidence of OHCA increased in 2020 in Pennsylvania relative to 2019. We hypothesized that avoidance of care would lead to decreased neurologically intact survival rates, fewer transports, and lower rates of bystander cardiopulmonary resuscitation (CPR) in the COVID era.

**Methods**: A total of 13,346 cases were reviewed from the Cardiac Arrest Registry to Enhance Survival (CARES) database. Descriptive statistics and appropriate comparative statistical techniques were performed under the guidance of professional statistician.

**Results**: The number of agencies in both 2019 and 2020 were the same and the median arrests per agency remained consistent. There was no significant difference in total number of cases of OHCA. We found a 3.3% increase in cardiac arrests at home in 2020, with fewer arrests in public places. There was no change in the percentage of witnessed cardiac arrests (53.2% vs 53.3%) or bystander CPR initiation (31.3% vs 31.0). Cardiac arrest outcomes had several significant changes. In 2020 vs 2019 there were fewer cases of sustained return of spontaneous circulation (ROSC) (68.0% vs 70.7%), and patients were less likely to be transported (68.6% vs 59.7%). Fewer patients survived to hospital admission (28.5% vs 24%), and more patients who survived to hospital admission were pronounced dead in the hospital in 2020 compared to 2019 (50.0% vs 42.4%). Among patients surviving to discharge, a greater percentage in 2020 had a cerebral performance category (CPC) of 1 or 2 compared to 2019 (84.5% vs 79.3%).

**Conclusion**: The increase in arrests at home is unsurprising considering pandemic related restrictions on public spaces. The absence of change in bystander CPR was an unanticipated result, however this may reflect low incidence of bystander interventions at baseline. We found an expected increase in field termination. We attribute this to guidelines regarding cardiac arrest management in suspected COVID‐19 patients published by Pennsylvania Department of Health (DOH) Bureau of Emergency Medical Services (EMS) (5) and the American Heart Association (4).

## 23 | How the COVID‐19 Pandemic Affected Different Specialties When it Came to Sleep Quality

**Palladino A, Deeb M, Stoltzfus J, Morley K, Patterson R, Stankewicz H**

**St. Luke's University Health Network, Easton, PA**

**Study Objectives**: The COVID‐19 pandemic affected healthcare professionals across many specialties. This study provides a look into how sleep quality was affected during the pandemic across different primary specialties.

**Methods**: The study was conducted through a survey via survey monkey which was sent to health care professionals of a healthcare network in eastern Pennsylvania. The study was sent to residents in training, fellows, nurses, attending physicians, as well as advanced practitioners in specialties including Emergency Medicine, Family Medicine, Internal Medicine, and Critical Care. The survey was sent out on January 11th, 2022 and remained open over a 2‐week period. All data collected in this study was anonymous. The responses to the survey questions about wellness were measured as 1 being not at all true to 5 being completely true. Several categories were compared to distinguish the providers from one another in addition to specialty, including age, with whom the provider lives, and the average number of clinical hours worked a month. Collectively, there were 267 surveys received of which 125 are male, 141 are female, and 1 prefers not to identify. 34 were Critical care, 147 were Emergency Medicine, 28 were Family Medicine, and 48 were Internal Medicine (Figure 1).

**FIGURE 1:**
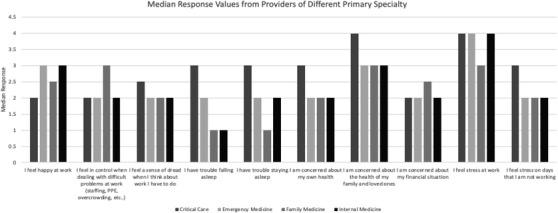
Median response values from health care professionals of different primary specialties.

**Results**: Significant differences occurred across primary specialties for 2 questions with clinical significance of p<.05. When asked “I have trouble falling asleep” there was a significant difference between specialties Critical Care vs Family Medicine p<.001. Critical Care vs Internal Medicine p = .002. Emergency Medicine vs Family Medicine p = .004. “I have trouble staying asleep” When asked the above question there was a significant difference between Critical Care vs Family Medicine p = .002 and Emergency Medicine vs Family Medicine p = .003.

**Conclusion**: The COVID‐19 pandemic affected health care professionals' sleep quality. Based on our study, the results indicate there is increased difficulty falling asleep and staying asleep among Critical Care and Emergency Medicine professionals compared to other specialties, particularly Internal Medicine and Family Medicine. The decrease in sleep quality experienced during the pandemic can potentially affect professionals' overall care.

## 25 | Early Findings and Barriers from the Construction of a Patient‐Empowering Implementation Framework in the Emergency Department

**Vyas N, Shahan J, Kraus C**

**Geisinger Commonwealth School of Medicine, Scranton, PA**

**Study Objectives**: Implementation frameworks are intended to facilitate adoption of evidence‐based practices and processes (EBPs) to bedside care. Geisinger's ProvenCare® model utilizes EBPs and standardizes algorithms to optimize patient outcomes. The program has been used for chronic obstructive pulmonary disease (COPD), bariatric surgery, total hip replacement, and coronary artery bypass grafting. Recently, ProvenCare® has been expanded to include patients with a diagnosis of pneumonia (PNA), including an emergency department (ED) to home component to optimize value for patients in an integrated health system. The objective of this study is to describe the initial experience, including barriers to implementation, with the ProvenCare® Pneumonia (PNA) ED to home (EDTH) program.

**Methods**: ProvenCare® PNA (Figure 1) assigns patients to hospitalization or EDTH, leveraging non‐hospital resources (eg, care management, remote monitoring) to avoid hospitalization using clinical judgment combined with objective risk stratification tools (eg, pneumonia severity index/PSI). Care algorithms aim to standardize care, including antibiotic stewardship. EDTH patients are discharged with a PNA kit, including pulse oximetry, and close follow‐up with care management and home care resources.

**FIGURE 1:**
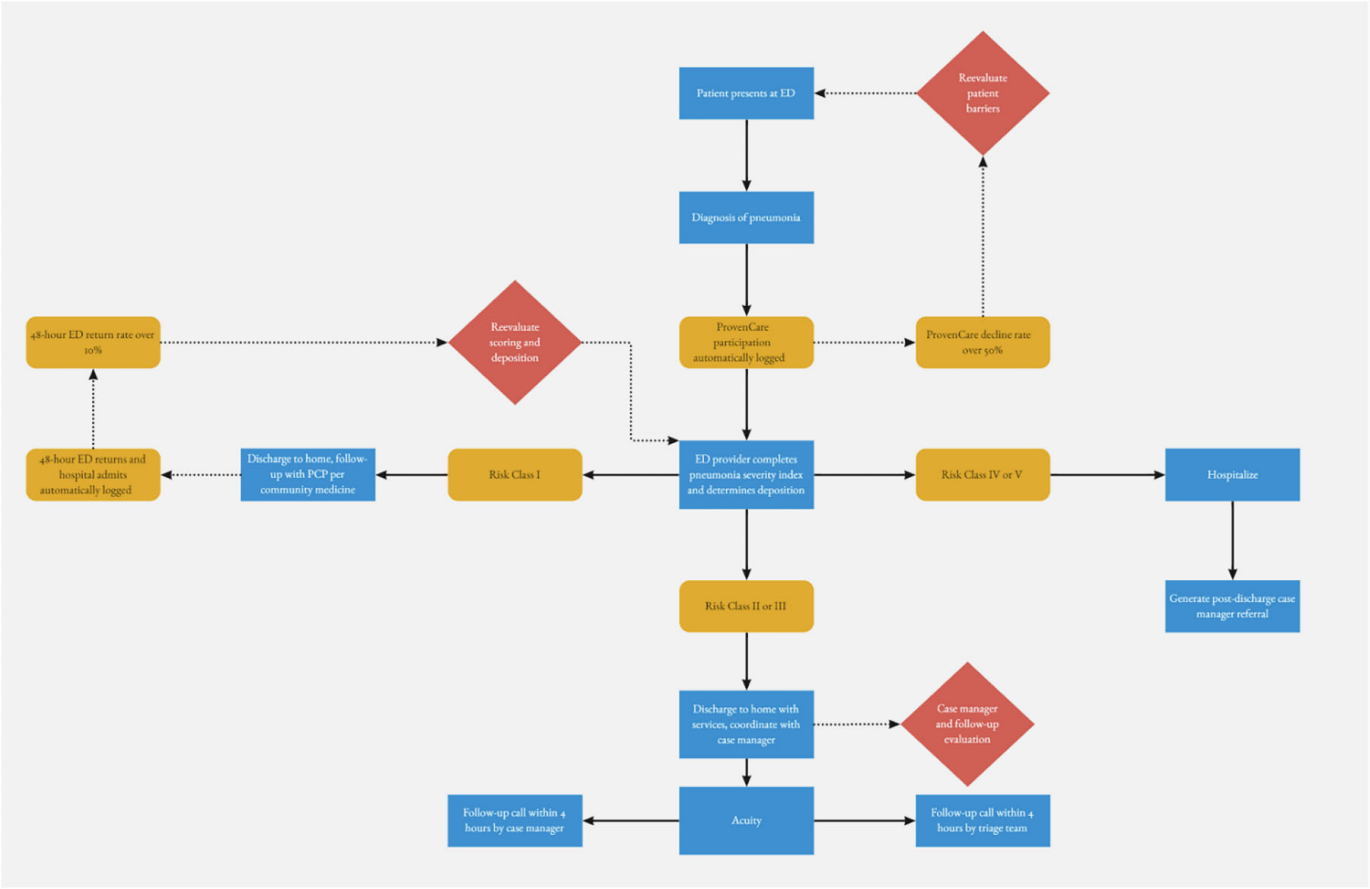
ProvenCare® PNA flow. Abbreviation: PNA, pneumonia.

**Results**: ProvenCare® PNA has enrolled 911 patients. The mean age is 68.4 years (SD = 18.4). 897 patients (98.5%) were hospitalized. 2 patients (<0.1%) returned to the ED, and one was re‐hospitalized. 81 patients (8.9%) tested positive for COVID‐19. The mean hospital length of stay for patients with COVID‐19 was 7.6 days compared to 5.8 days for patients without COVID‐19.

**Conclusion**: Implementation of an evidence‐based, resource‐dedicated EDTH program for patients with PNA is challenging. Most patients were hospitalized. Some patients with PNA diagnosis had COVID. Barriers to implementation include defining pneumonia diagnoses, adapting inpatient order sets, and showing financial and quality measure outcomes. Future research should focus on adaptations of the model with a larger sample of patients to ensure sustainability and desired outcomes.

## 26 | Intravenous Acetaminophen May Reduce Opioid Use for Pain in the Emergency Department

**DelBianco J, Deutsch A, Sharpe K, Laskosky J, Koons L, Beauchamp G, Katz K**

**Lehigh Valley Health. Network, Bethlehem, PA**

**Study Objectives**: Acute pain is a leading reason for Emergency Department (ED) evaluation. In 2018, there were 130 million ED visits for painful conditions. Also in 2018, 46,802 Americans died from an opioid‐related overdose in the ongoing epidemic. Therefore, providing effective non‐opioid analgesics in the ED is critical. Oral acetaminophen (APAP) is commonly administered in the ED but is limited to patients tolerating oral intake. Intravenous (IV) APAP provides significant pain reduction parenterally. For example, IV APAP reduces the need for additional post‐operative opioid administration and shows similar efficacy to IV morphine in treating renal colic pain. The purpose of this quality assessment project was to evaluate the safety of IV APAP, frequency of opioid use in patients receiving IV APAP and compliance with an ED IV APAP protocol.

**Methods**: This project included all patients who received IV APAP in the ED of a tertiary care, level I trauma center during a three‐month period. The protocol required ED patients be, nothing by mouth, 18 years or older, and receive a singular 1000 mg dose. Adverse reactions within 24 hours of IV APAP use, ED length of stay (LOS), and administration of opioids within four hours of IV APAP were all tracked. This quality assessment project was Institutional Review Board exempt.

**Results**: Ninety‐four patients received IV APAP. All patients received a 1,000 mg dose. One patient received more than one dose, but this patient had a 22‐hour ED LOS. Two patients received oral medications within one hour of IV APAP (one received an antacid, and the other received carbamazepine and lamotrigine). An opioid was administered to 22 of the 94 (23.4%) patients during the four‐hour protocol period. No adverse reactions were reported.

**Conclusion**: The results show excellent compliance with the protocol. IV APAP was safe and well‐tolerated. Notably, only 23.4% of the patients received an opioid within four hours of IV APAP. According to data from the National Center for Health Statistics, an opioid was given or prescribed for 30.9% of pain‐related ED visits nationally. The rising incidence of opioid use disorder and opioid overdose deaths involves both illicit and prescribed opioids. IV APAP can be safely and effectively utilized as an analgesic and lessen ED opioid use.

## 27 | How the COVID‐19 Pandemic Affected Different Ages Among Providers Regarding Perceived Stress Level in the Workplace

**Palladino A, Deeb M, Stoltzfus J, Morley K, Patterson R, Stankewicz H**

**St. Luke's University Health Network, Easton, PA**

**Study Objectives**: The wellness of providers and all members of the healthcare team is important for the holistic care of the patient and their loved ones. The COVID‐19 pandemic affected the wellness of providers in the healthcare field across America. This study provides insight into the degree with which each provider felt they were impacted, both positively or negatively.

**Methods**: This study was conducted through a survey sent out via survey monkey to providers of a health network in eastern Pennsylvania. Included in this study were residents in training, fellows in training, nurses, attending physicians and nurse practitioners/physician assistants in specialties including Emergency Medicine, Family Medicine, Internal Medicine, and Critical Care. The start date was January 11th, 2022 and the survey remained open for a 2‐week period. All surveys were anonymous. Several categories, in addition to specialty, were compared. These included age (20‐29, 30–39, 40–49, 50–59, and ≥60 years old), with whom the provider lives, and the average number of clinical hours worked per month.

**Results**: Collectively, there were 267 surveys received of which 125 are male, 141 are female, and 1 prefers not to identify. Results indicate a disparity between providers of different age groups regarding the survey question about wellness “I feel a sense of dread when I think about the work I have to do.” This disparity is specifically evident between age groups of 20–29 year olds versus ≥ 60 year olds (p‐value <.001), 30–39 year olds versus ≥ 60 year olds (p‐value < .001), and 40–49 year olds versus ≥ 60 year olds (p‐value = .002). The responses to the survey questions about wellness were measured as 1 being not at all true to 5 being completely true. Figure 1 indicates the median responses to the wellness questionnaire across the different age groups.

**FIGURE 1:**
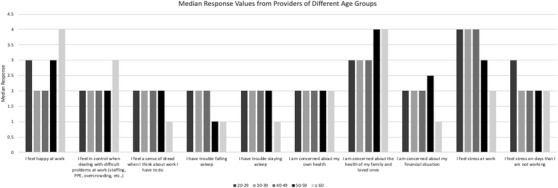
Median response values from providers of different age groups.

**Conclusion**: The pandemic played a role in the overall perceived stress level in the healthcare workplace among providers of different ages. Based on our study, the results indicate there is increased dread when “thinking about the work that needs to be done,” among younger providers (younger than 60 years old). The perceived sense of dread over the difficulties experienced in the pandemic, specifically among younger providers, can potentially negatively affect the providers in ways that should prompt further investigation.

## 30 | Expanded Use of Point of Care Ultrasound Curriculum for Attending Physicians

**Schultz C, Warren H, Roth K, Paulson C**

**Lehigh Valley Health Network, Bethlehem, PA**

**Study Objectives**: Ultrasound education is highlighted in the American College of Graduate Medical Education core curriculum for the emergency medicine residents, yet there is a gap in the desired goal of having all attending emergency physicians credentialed in the performance of expanded point of care ultrasound applications. The goal of this curriculum was to maximize the number of attending physicians credentialed in expanded point‐of‐care ultrasound (POCUS) applications.

**Methods**: A rapid education event (REE) to maximize the learning of the intended content was developed for the attending physician. The 4‐hour curriculum focused on four objectives as follows: 1) demonstration of lung ultrasound in the dyspneic patient, 2) defining expanded cardiac POCUS, 3) defining the E‐Fast exam, and 4) defining the protocol of scanning the abdominal aorta. A pre‐test was completed, then live didactic content was delivered to the participants. This was followed immediately with a hands‐on practice session with SPs, a direct observation grading of procedure by faculty and then a written post‐test. Our goal was for 90% of the participants to pass a post‐test with a score of 80% or higher. Additionally, we set out to have 100% of the participants complete a supervised POCUS.

**Results**: Thirteen attending physicians voluntarily participated. Twelve participants were emergency medicine and 1 was emergency and pediatric medicine. Sixty‐two percent (N = 8) were male, and thirty‐eight percent (N = 5) were female. The mean pre‐test score was 8.385/12 or 69.8% and the mean post test score was 9.62/12 or 80%. Eighty‐three percent (N = 10) scored an 80% or greater on the post‐test. Sixty‐nine percent (N = 9) improved their pre‐test scores, 23% (N = 3) had no change in their score, and 8% (N = 1) had a decrease in their score. The majority (85%, N = 11) completed direct observation of the POCUS procedures, see Table 1 for an example observation rubric. Data was missing from 15% of the learners (N = 2) due to either abstention from observation or failure to submit their checklist. No learner required remediation for any of the exams that were proctored. Ninety‐two (N = 12) of participants felt they were likely to expand their use and teaching of ultrasound on shift after this REE. All the participants (100%) felt that after this course they could procedurally meet the objectives taught during their next POCUS procedure on shift.

**TABLE 1:** Example of direct observation rubric.

Evaluator: Participant: **Aorta Skills Assessment**	**Answer**	**Rating: Satisfactory (2), Incomplete (1), Fail (0)**	Date: **Key**
Indication (verbalizes)	Abdominal pain Hypotension		Satisfactory: Lists at least one indication for an aorta POCUS Fail: Cannot list an indication for an aorta POCUS
Correct probe	Curvilinear or phased array		Satisfactory: Lists at least once correct probe Fail: Cannot identify a correct probe
Views required	Proximal, mid, distal and bifurcation in both transverse and longitudinal planes. Presence or absence of aneurysm should be documented. Views of the celiac artery, SMA and bifurcation are helpful but not required.		Satisfactory: Lists all 3 anatomical views required and verbalizes the need for 2 planes Incomplete: Lists < 3 views required or does not verbalize the need for 2 planes Fail: Cannot identify any of the 3 appropriate views
Documentation Required	Interpretation should note presence of absence of sonographic evidence of aneurysm and diameter if present.		Satisfactory: Lists the correct diameter interpretation of an aneurysm (>3 cm) Fail: Cannot verbalize the aneurysm sizing

Comments: (list all reasons for incomplete or fail).

**Conclusion**: While most of the participants were able to meet course threshold scores, the objective of 90% of participants passing the post‐test with greater than 80% was not achieved. However, nearly 2/3 of the participants were able to show improvement from pre‐test scores. The majority of participants did complete a directly observed procedure by faculty for the lung, echo, and Focused Assessment with Sonography for Trauma (FAST) exams, yet the goal of attaining 100% direct observation of each procedure by each participant was not completed. Evaluating learner preferences for being observed and more optimal data collecting strategies might improve these outcomes in future endeavors. Future follow up data is to be collected to see how this REE has impacted the participants credentialing in expanded applications and if it has changed billing practice.

## 32 | Emergency Medical Services Clinical Dashboard: Analyzing the Prehospital Care of Patients

**Huang E, Huff M, Harrison B, Bear E, Sharkazy J, Kane B, Wheel K, Stirparo**

**Lehigh Valley Health Network, Bethlehem, PA**

**Study Objectives**: Emergency medical services (EMS) play a key role in determining the overall quality of trauma care. Quality metrics for inpatient trauma care have been shown to improve patient outcomes. A statistically based performance improvement database for prehospital EMS care may have similar results. Our aim was to create a database for statistical analysis of prehospital care with regards to cervical spine immobilization, prolonged scene times, and splint application for long bone fractures.

**Methods**: We reviewed EMS care episodes from January 2021 to December 2021, including 508 encounters from 21 EMS Services in Northeastern Pennsylvania. Charts were individually reviewed, and variables of interest were extracted, including EMS team type and traumatic mechanism. Key performance indicators (KPIs) were collected, including cervical collar application, splinting when indicated, scene delays (>20 min), and appropriateness of overall care. Descriptive statistics were used to analyze the results.

**Results**: In aggregate, 20.0% of encounters had scene delays. Of these delays, only 51.4% were appropriate. There was substantial variation in scene time among agencies, ranging from 4.6% to 40.9% of encounters with a scene delay (Table 1). Failure to apply an indicated c‐collar and failure to apply indicated splinting occurred in 17.0% and 9.5% of all encounters, respectively. Among individual EMS groups, the range of failure to apply indicated cervical collars was 11.1% to 24.4% and failing to splint when indicated ranged from 2.4% to 18.2%.

**TABLE 1:** Overview of year 2021 EMS data by team, describing the presence and appropriateness of a scene delay.

EMS	Total Frequency	Scene Time > 20 Minutes	Delay Appropriate	Delay Reason Unknown
**EMS Team Alpha**	65	13 (20.0%)	6 (46.2%)	7 (35.8%)
**EMS Team Beta**	42	16 (38.1%)	6 (37.5%)	10 (62.5%)
**EMS Team Gamma**	22	1 (4.6%)	0	1 (100%)
**EMS Team Delta**	107	13 (12.2%)	9 (69.2%)	4 (30.8%)
**EMS Team Epsilon**	48	5 (10.4%)	2 (40.0%)	3 (60.0%)
**EMS Team Zeta**	128	24 (18.8%)	11 (45.8%)	13 (54.2%)
**EMS Team Eta**	22	9 (40.9%)	6 (66.7%)	3 (33.3%)

Abbreviation: EMS, emergency medical services.

**Conclusion**: Significant variation exists in the practices and frequency of compliance with established EMS care components and process KPIs. As EMS teams are vital to the prehospital care of trauma patients, it is of the upmost importance to identify variation and improve performance measures by means of established best practices. Future work will pair high and low performers in individual categories to standardize practices among groups.

## Footnotes

1 Numbers below are not in sequential order, but correspond to the Abstract number on the 2023 William Spivey Research Abstract Sessions.

